# The Hitchhiking Effect of a Strongly Selected Substitution in Male Germline on Neutral Polymorphism in a Monogamy Population

**DOI:** 10.1371/journal.pone.0071497

**Published:** 2013-08-28

**Authors:** Junrui Li, Kristan A. Schneider, Haipeng Li

**Affiliations:** 1 Laboratory of Evolutionary Genomics, CAS Key Laboratory of Computational Biology, CAS-MPG Parter Institute for Computational Biology, Chinese Academy of Sciences, Shanghai, China; 2 Graduate School of the Chinese Academy of Sciences, Beijing, China; 3 University of Applied Sciences, Mittweida, Germany; 4 Department of Mathematics, University of Vienna, Vienna, Austria; Kunming Institute of Zoology, Chinese Academy of Sciences, China

## Abstract

Comparative genomic studies suggest that a huge number of genes that show the strongest evidence for positive selection in human are testis- or sperm-specific genes, which are possibly due to germline selection. We propose a novel selection model in which the germlines of heterozygous males in a monogamous population are under natural selection. Under this model, we study the dynamics of a strongly selected substitution in the male germline and its hitch-hiking effect on the preexisting linked neutral polymorphism. We show that the expected heterozygosity at the neural locus is reduced by 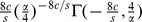, where *c* is the recombination rate between selected and neutral locus, *s* is selective coefficient of advantageous allele, 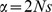 and *N* is diploid effective population size.

## Introduction

**Figure 1:**
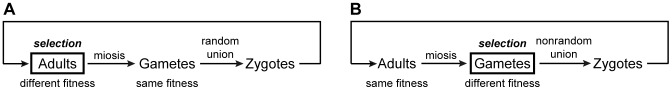
Model of positive natural selection in germline is different from that in individuals. (A) Individuals with different fitness are under selection but gametes have same fitness. (B) Individuals have same fitness but gametes with different fitness are under selection.

The hitchhiking effect [1] is commonly referred to a phenomenon that a selectively favored allele will change the frequencies of polymorphisms at linked loci on its way to fixation. This effect has been extensively studied in the last several decades [1]–[3] when the beneficial allele is codominant. Recently, Teshima and Przeworski examined the hitchhiking effect when the dominance coefficients of advantageous mutations are unknown [4]. To date, most models developed to characterize the hitchhiking effect assume that positive selection acts at the individual level through differential viabilities (Figure 1A). However, positive selection may also act on the male germline (Figure 1B) which might be common. In a comparative genomic study, based on the ratio of nonsynonymous substitutions per nonsynonymous site to synonymous substitutions per synonymous site (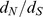), Nielsen *et al.* found a surprising large number of testis- or sperm-specific genes positively selected in the humans and chimpanzees [5]. A possible explanation for this is sperm competition [5].

Males produce tens of thousands of sperms, but only one of them can fertilize an egg. This severe competition implies potential positive selection that exert pressure on sperms activities in the spermatogenesis and fertilization process, for example, motility, acrosome reaction, penetration, apoptosis during spermatogenesis etc. Indeed, evidence for selection on the male germline has emerged taking the advantage of highly improved techniques in molecular biology. Several independent studies show genotype-dependent chances to fertilize eggs [6]–[8] and some reproductive proteins are identified as targets of positive selection [9]. As data on germline selection accumulates, it is critical to develop a model to characterize the dynamics of positive directional selection due to sperm competition, and its effects on linked neutral polymorphism.

This article considers the consequences of strongly-selected substitutions in the male germline on preexisting linked neutral ploymorphism. For simplicity, we make the following assumptions: (1) absence of viability selection, i.e., individuals with different genotypes have the same viability, and (2) monogamy, i.e., competition is only among different sperm haplotypes from the same heterozygous male, and there is no competition between sperms from different males.

## Two-Locus Model

We study a two-locus model that describes one selected and one linked neutral locus. At the selected locus, the ancestral allele is called *a* and the mutated beneficial allele *A*. According to our assumption, the viability of individuals with genotype *AA*/*Aa*/*aa* are the same and can be assigned as 1, which is different from the traditional dominant additive fitness model, a special case of arbitrary dominance model that is well studied by Teshima *et al.* [4], but sperms with allele *A* have a selective advantage over sperms with allele *a* and their fitness are assigned as 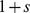, and 1, respectively. However, sperms from a homozygous male (*AA* or *aa*) have no advantage over other sperms from the same individual since they are identical. Thus their fitnesses are set to 1. The alleles at the neutral locus are denoted by *B* and *b*. We assume that a beneficial mutation *A* arise at time 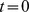 and replaces allele *a* subsequently. The fixation process of alleles *A* may alter heterozygosity levels at the linked neutral locus.

To analyze this model, we follow Ohta and Kimura's treatment that separated the dynamics of the selected locus and that of the neutral locus [2]. Afterwards, we consider only the frequency trajectory of the beneficial allele, which is acceptable when selection is so strong that the dynamics can be treated deterministically [10]. Furthermore, we adopt a modified moment-analysis method [3] to study the effect of the beneficial allele on the neutral locus.

## Results

### Frequency trajectory of beneficial allele

Assume the frequency of individuals with genotype *AA*, *Aa* and *aa* in the current generation is *P*, 2*Q*, and *R*, respectively, where 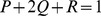. In terms of genotype frequencies, the allele frequencies *p* of *A* and *q* of *a* are as follows:
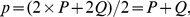
(1a)

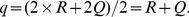
(1b)Note that 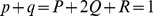.

According to the sperm competition model we used, we can calculate the frequencies of allele *A* and *a* in the next generation. In total, there are nine possible mating pairs (Table 1). Since competition only occurs among sperms, there will be no selection at the individual level. So mating will be random and these matings take place in proportion to the genotype frequencies. For example, the proportion of *Aa* and *AA* matings is 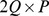. The frequencies of all the nine mating pairs are given in Table 1.

**Table 1:** Genotype frequency in next generation.

male×female	freq. of mating pair	freq. of *AA* zygotes	freq. of *Aa* zygotes	freq. of *aa* zygotes
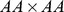	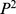	1	0	0
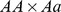	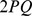	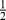	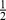	0
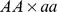	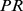	0	1	0
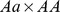	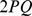	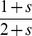	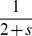	0
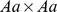	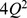	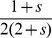	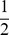	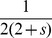
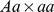	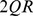	0	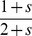	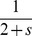
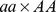	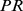	0	1	0
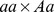	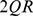	0	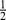	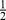
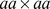	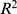	0	0	1

First two columns are all nine possible mating pairs and their frequencies. Last three columns are frequencies of zygotes produced by different mating pairs under germline selection.

Recall that, competitions only happen in the gametes produced by the heterozygous male. Thus, a mating of male *Aa* with female *AA* produces proportionally 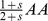 and 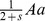 zygotes, while the mating of male *AA* with female *Aa* produce 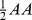 and 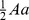 zygotes. The frequency of zygotes produced by all possible mating pairs is listed in Table 1.

The genotype frequencies of *AA*, *Aa*, and *aa* zygotes at the next generation are denoted as 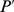, 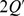, and 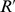, respectively. These frequencies can be calculated as follows:
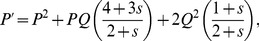
(2a)

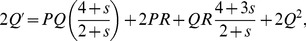
(2b)

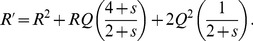
(2c)


Then, the frequencies of *A* and *a*, which are denoted as 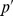 and 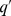, respectively, can be calculated from genotype frequencies:
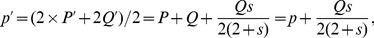
(3a)

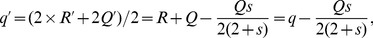
(3b)where *p* and *q* are the allele frequencies given in Equation (1).

From Equation (3a), we obtain the frequency change of the *A* allele after one generation of germline selection,
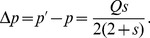
(4)


In the traditional positive selection models, random union of gametes guaranties Hardy-Weinberg equilibrium, which means
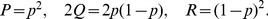
(5)


But in the germline selection model, gametes from the same heterozygous male have different fitness, which leads to non-random union of gametes. Thus genotype frequencies deviate from the expectation of Hardy-Weinberg principle. However, we can use Hardy-Weinberg proportions as a good approximation to calculate frequency change of allele *A*:
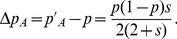
(6)The difference between equation (4) and (6) arises from frequency differences of heterozygotes (*Aa*) after one generation of germline selection. Here, without loss of generality, we use frequencies of heterozygote *Aa* in (*i*+1)-th generation as an example to show that differences between frequencies of *Aa* heterozygotes calculated from equation (4) and (6) are a first order infinitesimal item of *s*, i.e., *O*(*s*).

Equation (6) yields the frequency of allele *A* after one generation of germline selection as
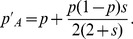
(7)Since, 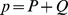, we can derive the deviation between 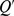 and 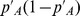 to be
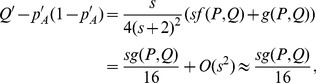
(8)a first order infinitesimal item of *s*, where 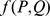 and 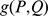 are polynomials of *P* and *Q*.

**Figure 2:**
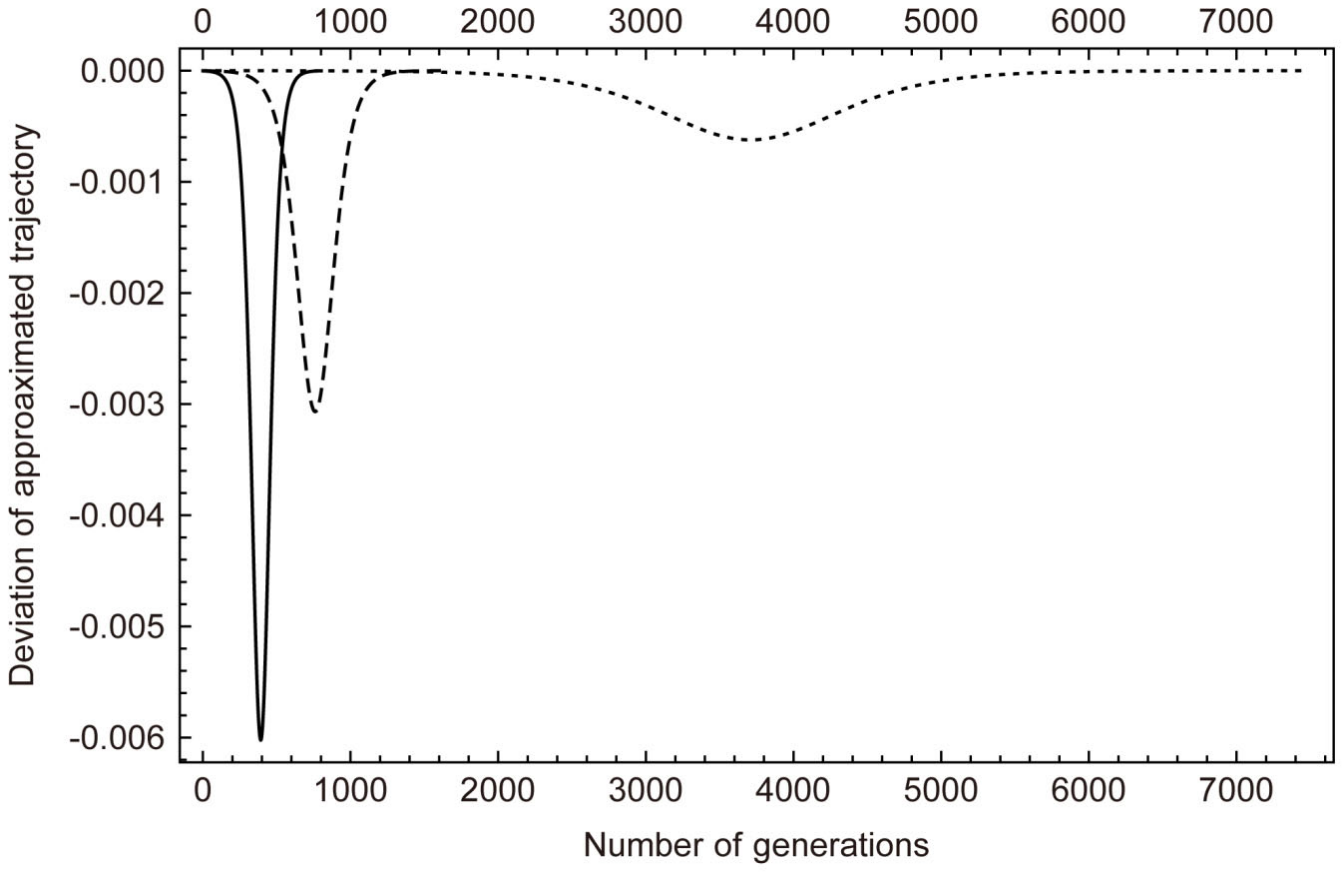
Deviation between HW approximated and real frequency trajectories of allele *A*. Solid line, dashed line, and dotted line are for selective coefficients 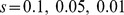, respectively.

Then, equation (4) can be written as
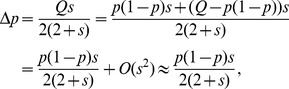
(9)which means that Hardy-Weinberg frequencies are a good approximation. The deviation between frequency trajectories that are calculated iteratively by Equation (4) and Equation (9) is demonstrated in Figure 2. Given selective strength *s*, the absolute value of this deviation is positively correlated with the frequency of heterozygotes *Aa* (Figure S1). That means the more heterozygotes, the larger the deviation, and the deviation attains its maximum when the frequency of heterozygotes is maximized (results not shown). Of note, the maximum deviation increases with increasing selective coefficients *s*. However, the deviation is still very small even when *s* is as large as 0.1 (Figure 2). Moreover, difference in fixation times of these two frequency trajectories are also negligible (results not shown). Therefore, Equation (9) is a good approximation for the frequency trajectory of allele *A*.

If the selective advantage of allele *A* is large, the frequency change of allele *A* can be treated deterministically as long as its frequency is not very close to either 0 or 1 [11]. Given 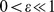, the frequency of allele *A* at time *t* can be approximated by 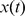 that satisfies the differential equation:
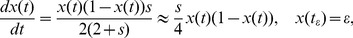
(10)where we make a further approximation using the Taylor expansion of 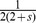 and ignoring terms of order 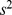 and higher.

The solution of this ordinary differential equation (ODE) is
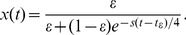
(11)


For convenience, by the substitution 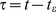, the time it takes for allele *A* to reach quasi-fixation, i.e., the time needed to increase from frequency 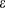 to 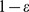, can be calculated to be
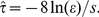
(12)


### Hitchhiking effect on heterozygosity

In order to study the effect of the selected locus on the neutral one, we adopt the method of Stephan *et al.* [3], which is modified from Ohta and Kimura's moment-analysis method [2]. Ohta and Kimura divide the population into two parts: one part contains chromosomes carrying the advantageous allele *A* and the other part carrying the disadvantageous allele *a* [2]. Let 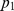 be the frequency of allele *B* among chromosomes carrying *A*, and 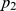 be the frequency of *B* among chromosomes carrying *a*. Then the frequency of allele *B* can be expressed by 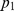 and 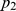 as
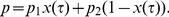
(13)


Following Stephan *et al.*'s method [3], which distinguishes the situations that the beneficial mutation occurs on a *B*- or *b*-carrying chromosomes, we calculate a weighted expectation for an arbitrary polynomial function *f* of 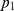 and 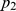 as
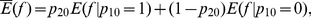
(14)where 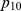 is the frequency 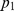 at time 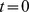, and 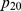 is the frequency 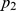 at time 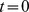. The former equals either one or zero, depending on whether the beneficial mutation occurred on a *B* or *b* background.

The expected heterozygosity *H* at *B*/*b* locus is defined by
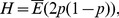
(15)where *p* is the frequency of allele *B* (Equation (17)).

**Figure 3:**
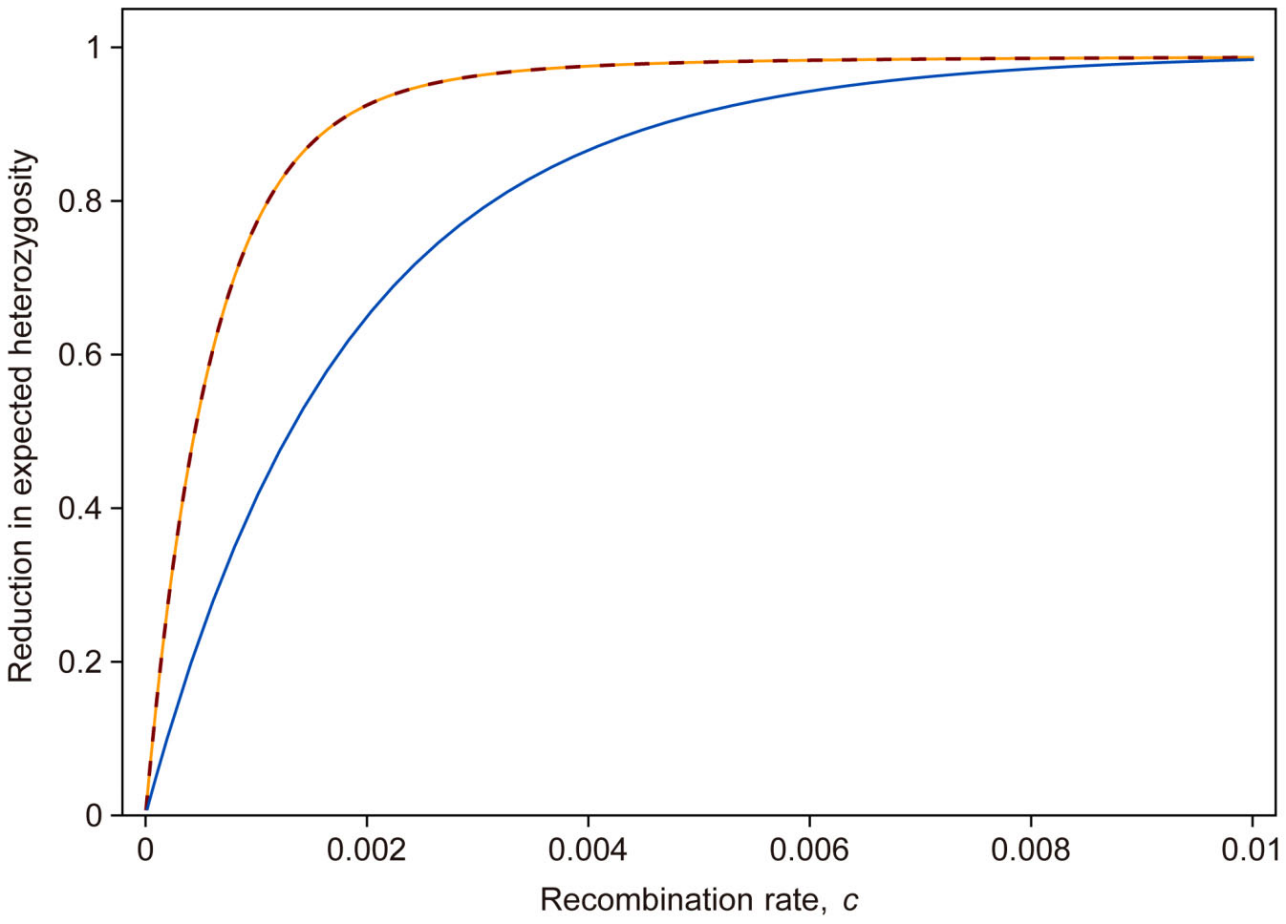
Reduction in expected heterozygosity at the end of selection process. Solid orange line shows the expected heterozygosity calculated from Equation (16). Dashed line is the approximated result (Equation (17)). Solid blue line demonstrates the reduction in heterozygosity according to result of Stephan *et al.* (1992). We assumed 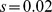.

A series of differential equations are derived and solved approximately following Stephan *et al.*'s method [3], and then we obtained the reduction in expected heterozygosity at the end of selection:
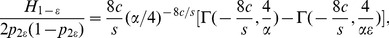
(16)where 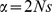, *c* is recombination rate between selected locus and neutral locus, 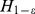 and 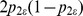 are the expected heterozygosities at time when the frequencies of the allele *A* are 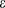 and 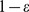, respectively. Note, that this is exactly the same form as the one derived by Stephan *et al.* [3] with *s* replaced by 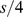. Intuitively, this is not too surprising, because selection occurs only in heterozygote males. Since males account only for half of the population, and at most half of the males are heterozygous, selection is roughly four times less efficient than in a corresponding viability selection model. The reduction in expected heterozygosity of these two models is demonstrated in Figure 3.

If 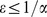, then the Equation (16) can be approximated as
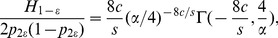
(17)which imply that reduction in expected heterozygosity is only weakly dependent on 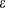. Figure 3 shows that this is a very accurate approximation for various choices of *c*.

## Discussion

Exploring alternatives to adaptive evolution driven by differential viabilities, we proposed a model in which sperms from a heterozygous male are under natural selection. This model is different from the classic models since the male germline rather than individuals are selected. Notably, this model is also distinct from fertility selection [12]–[14] where different mating pairs have different fertility, as well as sexual selection [15], [16] in which selection functions through asymmetrical mating preferences. A similar model which is mentioned as “meiotic drive” has been study by Chevin and Hospital [17], however, their model sticks to hitchhiking effect results from non-random segregation of chromosomes during meiosis rather than germline competition after meiosis. Thus, their results introduce an additional factor that is related to recombination rate to describe hitchhiking effect, while our results related to selective strength. Compared to other sperm competition models [18]–[20] where ployandrous populations are considered, this model mainly focuses on sperm competition in heterozygous males, and it is more suitable to describe the evolutionary dynamics of germline selection in monogamous population, for example the testis- or sperm-specific genes in human population [5]. Interestingly, recently evolved new genes are often testis-specific as documented by Vinckenbosch *et al.* [21], and Zhang *et al.* [22]. Moreover, these genes are often associated with strong adaptive signals [22]. As argued by Meiklejohn and Tao, such new genes may originate under the pressure of meiotic drive [23]. However, as the model discussed in this manuscript, such genes may adaptively emerge under sperm competition.

Here, we studied the dynamics of a strongly selected substitution in male germline and its effect on the preexisting linked neutral polymorphism. Due to the selection on male germline, random union of sperms and eggs are disrupted. Thus, genotype frequency and allele frequency are no longer in Hardy-Weinberg equilibrium. However, the deviation of genotype frequencies from Hardy-Weinberg proportions are negligible (Figure 2) and we found that Hardy-Weinberg frequencies calculated from genotype frequencies can still produce a good approximation for allele frequencies.

The dynamics of the selected allele and its effect on linked neutral polymorphism are similar to the results derived by Stephan *et al.* [3], except that the selective coefficient in our formulas is 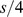 rather than *s*, which means that germline selection is weaker than individual selection, given the same selection coefficient. This is reasonable because selection only occurs in heterozygous males. However, the selective strength in germlines may usually be much larger than that in individuals, which results in faster evolution of reproductive genes [24]–[26].

Notably, the hitchhiking effect of a beneficial mutation selected in the male germline is approximately identical to that of a beneficial mutation under viability selection in the absence of dominance, however, having just a quarter of the selective advantage. Hence, in genome-wide scans for traces of selection, positively selected targets in the male germline might be incorrectly inferred as candidates under moderate viability selection. Even further, in such situations between-population comparison studies designed to verify adaptive evolution might not provide evidence for viability selection and hitchhiking patters might be incorrectly explained by demographic effects. Hence, in populations in which germeline selection could potentially occur, experimental setups need to be adjusted accordingly to correctly infer the mechanisms of selection.

As a basic model for germline selection, the model can be extended to some more complex models. For example, fitness of individuals can also be considered in the model. Several recent studies about a substitution in fibroblast growth factor receptor 2 (*FGFR2*) in the male germline showed that a selective germline advantage leads to unexpected high mutant prevalence, although this substitution causes defects to the descendants [27], [28]. It indicates that the substitution is positively selected in germlines, but negatively selected in individuals, which could lead to overall balancing selection. Hence, models combining these two aspects may give a better prediction for diseases and worth further investigations. Notably our model assumes a monogamous population. However, it can also be extended to polyandry populations where females mate with different males in a short time period, which certainly leads to more severe sperm competition. Such phenomenon can be frequently found in social insects, for example bees [29]. In such cases the reduction of linked neutral polymorphism may accordingly be more severe.

## Supporting Information

Figure S1 **Correlation between the deviation and HW frequency of heterozygotes.** Horizontal axis is the number of iteration. HW frequency of heterozygotes is demonstrated by the solid line and left vertical axis. The deviation between HW approximated and the real frequency trajectories of allele *A* is demonstrated by the dashed line and right vertical axis. We assumed *s* = 0.1.
(TIF)
