# Supplementary figures and images for: The Hitchhiking Effect of a Strongly Selected Substitution in Male Germline on Neutral Polymorphism in a Monogamy Population
Source: PLoS One. 2013 Aug 28;8(8):e71497. doi: 10.1371/journal.pone.0071497 (PMC3756016; doi:10.1371/journal.pone.0071497)

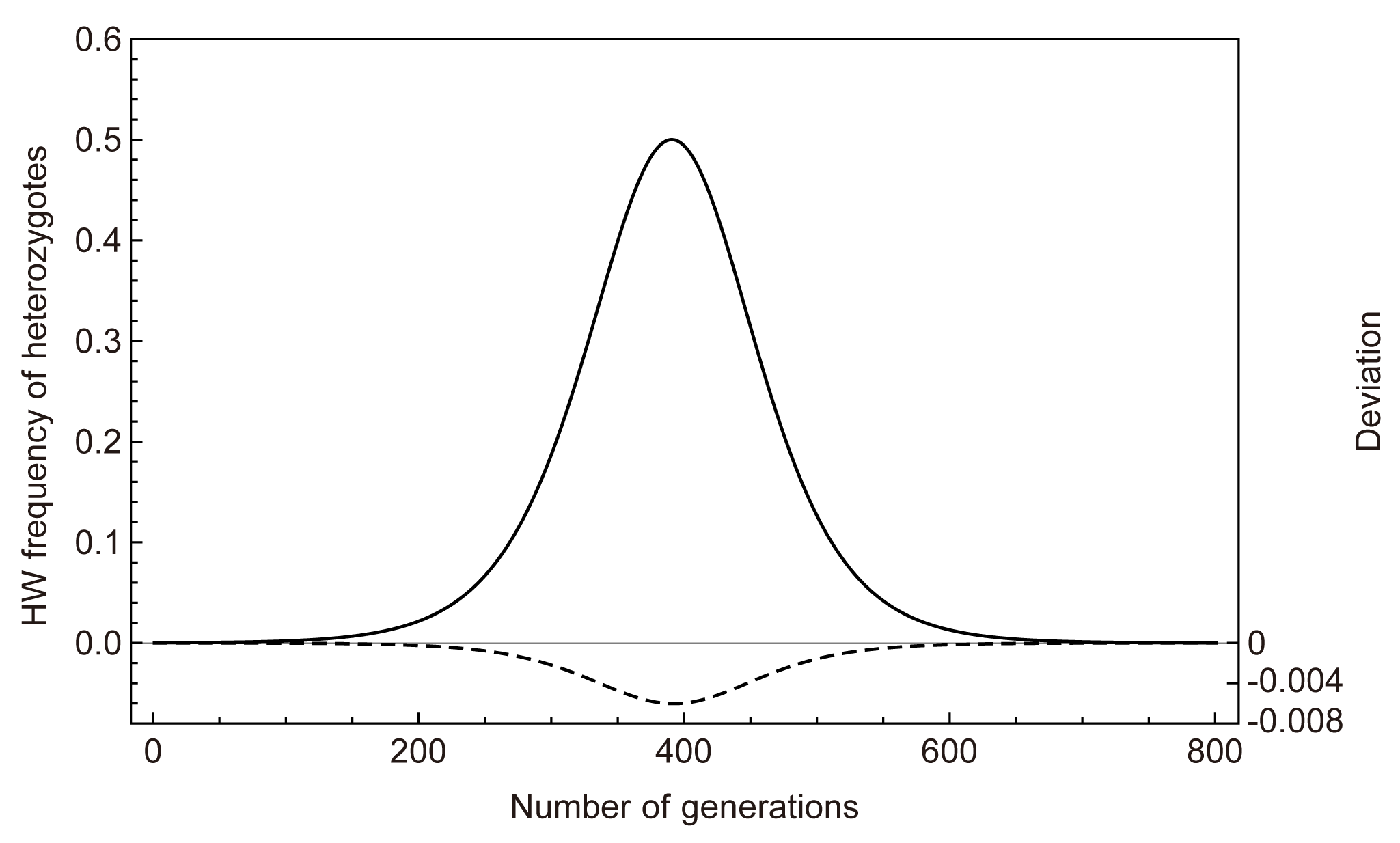

Supplement: Figure S1 — Correlation between the deviation and HW frequency of heterozygotes. Horizontal axis is the number of iteration. HW frequency of heterozygotes is demonstrated by the solid line and left vertical axis. The deviation between HW approximated and the real frequency trajectories of allele A is demonstrated by the dashed line and right vertical axis. We assumed s = 0.1. (TIF) [file pone.0071497.s001.tif]
